# Quality of life and function after rectal cancer surgery with and without sphincter preservation

**DOI:** 10.3389/fonc.2022.944843

**Published:** 2022-10-21

**Authors:** Emmanouil P. Pappou, Larissa K. Temple, Sujata Patil, J. Joshua Smith, Iris H. Wei, Garrett M. Nash, José G. Guillem, Maria Widmar, Martin R. Weiser, Philip B. Paty, Deborah Schrag, Julio Garcia-Aguilar

**Affiliations:** ^1^ Department of Surgery, Memorial Sloan Kettering Cancer Center, New York, NY, United States; ^2^ Department of Surgery, University of Rochester Medical Center, Rochester, NY, United States; ^3^ Department of Quantitative Health Sciences, Cleveland Clinic, Cleveland, OH, United States; ^4^ Department of Surgery, UNC School of Medicine, Chapel Hill, NC, United States; ^5^ Department of Medicine, Memorial Sloan Kettering Cancer Center, New York, NY, United States

**Keywords:** rectal cancer, LARS – low anterior resektion syndrom, patient reported clinical outcomes, quality of Life, MSKCC = memorial sloan–kettering cancer center

## Abstract

Despite improvements in surgical techniques, functional outcomes and quality of life after therapy for rectal cancer remain suboptimal. We sought to prospectively evaluate the effect of bowel, bladder, and sexual functional outcomes on health-related quality of life (QOL) in patients with restorative versus non-restorative resections after rectal cancer surgery. A cohort of 211 patients with clinical stage I-III rectal cancer who underwent open surgery between 2006 and 2009 at Memorial Sloan Kettering were included. Subjects were asked to complete surveys preoperatively and at 6, 12, and 24 months after surgery. Validated instruments were used to measure QOL, bowel, bladder, and sexual function. Univariable and multivariable regression analyses evaluated predictors of 24- month QOL. In addition, longitudinal trends over the study period were evaluated using repeated measures models. In total, 180 patients (85%) completed at least 1 survey, and response rates at each time point were high (>70%). QOL was most impaired at 6 and 12 months and returned to baseline levels at 24 months. Among patients who underwent sphincter-preserving surgery (SPS; n=153 [85%]), overall bowel function at 24 months was significantly impaired and never returned to baseline. There were no differences in QOL at 24 months between patients who underwent SPS and those who did not (p=.29). Bowel function was correlated with QOL at 24 months (Pearson correlation,.41; p<.001). QOL among patients who have undergone SPS for rectal cancer is good despite poor function. Patients with ostomies are able to adjust to the functional changes and, overall, have good global QOL. Patients with low anastomoses had lower global QOL at 24 months than patients with permanent stomas. Our findings can help patients set expectations about function and quality of life after surgery for rectal cancer with and without a permanent stoma.

## Introduction

Therapy for rectal cancer continues to evolve. Historically, regardless of tumor location, treatment included removal of the rectum and sphincter, resulting in a permanent stoma. With a better understanding of the recurrence patterns of rectal cancer, advances in surgical techniques, and more effective neoadjuvant therapy, sphincter-preserving surgery (SPS) is often feasible for low rectal cancers, and the need for a permanent stoma is less common.

Despite these advances, functional outcomes remain less than optimal (1). Bowel dysfunction that occurs after SPS can have a profound effect on quality of life (QOL) and lead to permanent disability, especially in patients who undergo neoadjuvant therapy and with a very low anastomosis (2).

Additionally, there is often significant aversion to stomas among patients in the preoperative setting. Having a stoma has been shown to have a negative effect on body image and sexual function and a positive effect on gastrointestinal problems (3). Life with a permanent stoma is assumed to be inferior to life with SPS. Even among specialty centers, there is significant variation in the rates of sphincter preservation for low rectal tumors, suggesting that uncertainty remains regarding who should have sphincter preservation (4).

Few studies have evaluated the comparative effectiveness of SPS with permanent stoma with respect to patient-reported outcomes (PROs). A systematic review which included nineteen studies with a total of 6453 patients concluded that it is not possible to draw a firm conclusion on postoperative QoL and body image following restorative versus non-restorative rectal cancer surgery (5).Post-treatment function and quality of life in patients with rectal cancer have been insufficiently studied (6).

The purpose of this study was to prospectively describe functional outcomes in patients undergoing curative resection for rectal cancer, with and without sphincter preservation, and to evaluate the effect of these functional outcomes on QOL. The identification of predictors and correlates of poor QOL preoperatively will enable patients to set realistic expectations and be more satisfied with the outcomes of their treatment.

## Methods

### Eligibility criteria

This study was approved by the Institutional Review Board at Memorial Sloan Kettering Cancer Center (MSKCC). A cohort of patients diagnosed with clinical stage I-III rectal cancer who underwent rectal cancer surgery at MSKCC between December 2006 and August 2009 was identified. Patients were eligible if they received their primary surgery at MSKCC, had no evidence of metastatic disease at the time of consent, and were able to complete surveys in English. Patients were excluded if they did not have surgery at MKSCC after consenting to the study, were found to have metastatic disease before completing the baseline survey, or underwent artificial sphincter placement.

Patients were classified as having undergone SPS or non-SPS on the basis of the intention of the initial surgery. Patients who underwent temporary diversion either with diverting ileostomy or diverting colostomy were considered to have undergone SPS. SPS procedures included low anterior resection (LAR) and LAR with coloanal anastomosis (LAR/CAA); non-SPS procedures included abdominoperineal resection (APR), Hartmann’s procedure, and proctectomy. Patients who underwent SPS with temporary diversion but did not have bowel continuity restored or who later had a permanent stoma placed were withdrawn from the study, as they underwent multiple procedures and had a markedly different experience from the rest of the cohort.

All operations were performed using the open approach by surgical oncologists specializing in colorectal cancer according to the standard techniques of total mesorectal excision and nerve preservation (7, 8). Minimally invasive techniques were not used for rectal cancer at the time of the study due to oncologic concerns of minimally invasive surgery at the time of the study. On the basis of *a priori* criteria, patients were withdrawn from the study if they (1) asked to withdraw, (2) missed two consecutive surveys, (3) developed a new primary cancer, (4) developed metastatic disease, or (5) died. Data collected on patients before withdrawal were kept and used in the data analysis.

### Survey variables

Subjects were asked to complete paper surveys preoperatively and at 6, 12, and 24 months after the completion of all surgical therapy. Patients who underwent temporary diversion completed surveys at 6, 12, and 24 months after restoration of bowel continuity. Surveys were mailed to patients at the appropriate times. Patients who did not return the survey were telephoned and mailed follow-up surveys two weeks later (9). The surveys incorporated validated tools to assess function and QOL. Bowel function was assessed at all time points using the Memorial Sloan Kettering Bowel Function Instrument (MSK BFI), which has been previously validated for rectal cancer patients (10). The MSK BFI includes 18 items, with 3 subscales and an overall bowel function score. On the basis of our previous work, a 4- to 5-point difference in BFI score is considered clinically significant. Sexual function was assessed preoperatively and at 12 and 24 months using the FSFI for women (11, 12) and the IIEF for men (13). Both the IIEF and the FSFI have validated cutoff points of a total score >1 standard deviation below the mean score of a normal population (14). To be able to evaluate both men and women in one analysis, rather than in two sex-specific models, we used these established cutoffs to create one binary global sexual dysfunction variable. For the FSFI, this cutoff was 25.2 (11, 12); for the IIEF, it was 42.9 (14). Bowel, bladder and sexual function were assessed using the EORTC QLQ-CR38 (15). In this report, we focus on the function-specific instrument measures (MSK BFI, IIEF, FSFI), for the evaluation of bowel and sexual function. The subscales of the EORTC QLQ-CR38 believed to be most strongly associated with bowel and sexual function were compared to the function-specific instrument scores to ensure that they corroborated (data not shown). Bladder function was evaluated using the EORTC QLQ-CR38 micturition subscale.

QOL was assessed based on the global QOL subscale of the EORTC QLQ-30, preoperatively and at 6, 12, and 24 months (16). A 5- to 10-point difference in global QOL score is considered to be clinically significant (17, 18). Subscales of the EORTC QLQ-C30 identified *a priori* were also evaluated.

Missing data is a universal issue in questionnaire-based surveys. Patients who did not complete a survey at one or more time points (attrition) were compared with patients who completed surveys at all time points. Patient demographic, tumor, and treatment characteristics that were abstracted from medical records were compared. We did not observe any clinically meaningful differences in these comparisons.

Another type of missing data occurs when a patient fills out a survey but does so leaving individual items incomplete. This type of missing data was dealt with in accordance with the recommendations published in the individual instrument manuals. If a patient did not complete >50% of survey items in each domain (bowel, bladder, sexual function, and overall health-related QOL), the composite score was set to “missing” (10, 11, 13, 15, 16).

### Statistical analysis

The primary outcome measure was global QOL at 24 months, using the EORTC QLQ-C30. Univariate associations between 24-month QOL and clinical and demographic features, as well as other functional measures, were assessed using Student’s t test or ANOVA as appropriate. On the basis of these findings, a multivariate regression model was constructed to identify factors that had an independent influence on QOL. Some variables were included that were deemed clinically important but not significant in univariate analysis. Similar analyses were conducted to evaluate 24-month bowel, bladder and sexual function.

In addition to the 24-month outcomes analyses, the trends of bowel, bladder, and sexual function were assessed for the study time points (baseline, 6, 12, and 24 months) using repeated measures models with a compound symmetry covariance structure. These models incorporate patient-level correlations and allow for the inclusion of all patients in the analysis, regardless of how many survey time points were completed (19, 20). For presentation, predicted values based on the model were generated and plotted with standard errors (20). For bowel function, only patients who underwent SPS were included in the analyses, because the items were relevant only to patients without a stoma; grouping non-SPS and SPS patients for all other analyses was deemed appropriate, given the small sample size of non-SPS patients. Sensitivity analyses including and excluding non-SPS patients were performed to evaluate the result of including these patients. All analyses were conducted using SAS version 9.1. P values <.05 were considered to indicate statistical significance.

## Results

### Study participants

**Figure 1:**
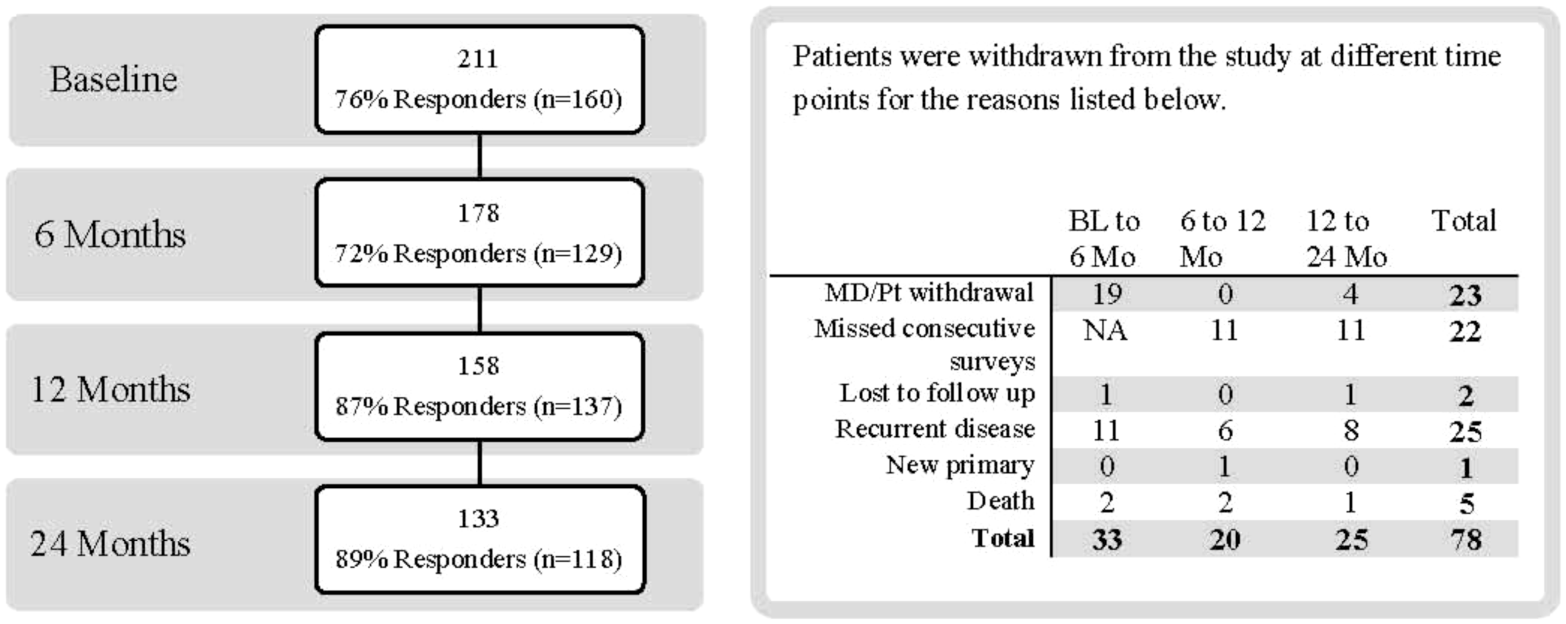
Response rates and withdrawals during the study. BL, baseline; MD, medical doctor; Mo, months; Pt, patient.

Two hundred eighty patients were approached for the study, of whom 229 consented to participate and 211 were deemed eligible. Of these, 76% completed the baseline evaluation (n=160). An additional 20 patients were included in the cohort who did not complete the baseline evaluation but did complete the 6-month evaluations. Thus, our cohort comprises 180 patients (85% of eligible patients). Our response rates ranged from 72% to 89% at the 4 time points. Because we were interested in evaluating QOL for rectal cancer survivors in the absence of metastatic or recurrent disease, patients were censored at the time of recurrence (n=25), a new primary cancer diagnosis (n=1), or death (n=5). Two patients did not return to the clinic, 23 withdrew, and 22 missed two consecutive surveys (**Figure 1**).

The clinical and demographic characteristics of the study cohort (n=180) are shown in **Table 1**. The majority of patients in our cohort were male (58%), relatively young (median age, 56), had low tumors (median, 7 cm from the anal verge), locally advanced stage disease (56%), and had been treated with neoadjuvant therapy (80%). Preoperative radiation treatment was common (63%). Of the 180 patients, 153 (85%) underwent SPS, and 27 (15%) underwent non-SPS, mostly APR (89%). Patients who underwent APR were older, had more-distal tumors, and were more likely to have received radiation, compared with patients who underwent SPS. Most patients who underwent SPS had temporary diverting ostomies (76%) and had an ostomy reversal at a median time of 6 months after the primary surgery. Of the 153 SPS procedures, 64% had coloanal anastomoses, 32% had pouch reconstructions, and 26% had handsewn anastomoses.

**Table 1:** Demographic characteristics.

Characteristic	Eligible patients (N = 211)	Patient cohort (N = 180)	No SPS (N = 27)	SPS (N = 153)
Sex				
Male	122 (58)	105 (58)	22 (81)	83 (54)
Female	89 (42)	75 (42)	5 (19)	70 (46)
Age at Surgery				
<55	97 (46)	84 (47)	10 (37)	74 (48)
55-74	98 (46)	84 (47)	13 (48)	71 (46)
≥75	16 (8)	12 (7)	4 (15)	8 (5)
Marital Status				
Single/Widowed	57 (27)	50 (28)	7 (26)	43 (28)
Married	154 (73)	130 (72)	20 (74)	110 (72)
Preop Stage				
I	34 (16)	27 (15)	3 (11)	24 (16)
II	42 (20)	35 (19)	9 (33)	26 (17)
III	116 (55)	100 (56)	11 (41)	89 (58)
Unknown	19 (9)	18 (10)	4 (15)	14 (9)
Postop Stage				
0	51 (24)	47 (26)	3 (11)	44 (29)
I	62 (29)	51 (28)	7 (26)	44 (29)
II	45 (21)	38 (21)	10 (37)	28 (18)
III	49 (23)	40 (22)	6 (22)	34 (22)
IV	4 (2)	4 (2)	1 (4)	3 (2)
Preop Therapy				
Chemo Only	33 (16)	30 (17)	0	30 (20)
Chemo/RT	127 (60)	112 (62)	21 (78)	91 (59)
RT Only	1 (0.5)	1 (0.6)	5 (19)	0
None/NA	49 (23)	37 (21)	1 (4)	32 (21)
Surgical Procedure				
APR	28 (13)	24 (13)	24 (89)	0
LAR	62 (29)	52 (29)	0	52 (34)
LAR Hartmann’s	3 (1)	3 (2)	3 (11)	0
LAR/CAA	114 (54)	98 (54)	0	98 (64)
TAE	3 (1)	2 (1)	0	2 (1)
TEM	1 (0.5)	1 (0.6)	0	1 (0.7)
SPS				
Pouch	54 (26)	49 (27)	0	49 (32)
Straight	122 (58)	101 (56)	0	101 (66)
NA	35 (17)	30 (17)	27 (100)	3 (2)
SPS – Anastomosis				
Handsewn	47 (22)	40 (22)	0	40 (26)
Stapled	129 (61)	110 (61)	0	110 (72)
NA	35 (17)	30 (17)	27 (100)	3 (2)
Diversion				
Colostomy	32 (15)	27 (15)	27 (100)	0
Ileostomy	133 (63)	116 (64)	0	116 (76)
None	46 (22)	37 (21)	0	37 (24)
Distance from Anal Verge, cm				
<5	42 (20)	34 (19)	15 (56)	19 (12)
5-10	122 (58)	106 (59)	10 (37)	96 (63)
≥10	47 (22)	40 (22)	2 (7)	38 (25)
Anastomosis Level, cm^a^				
≤4	70 (33)		N/A	61 (40)
>4	74 (35)		N/A	65 (42)
Surgeon				
1	41 (19)	35 (19)	10 (37)	25 (16)
2	1 (0.5)	1 (0.6)	0	1 (0.7)
3	26 (12)	16 (9)	2 (7)	14 (9)
4	19 (9)	19 (11)	3 (11)	16 (10)
5	40 (19)	38 (21)	4 (15)	34 (22)
6	84 (40)	71 (39)	8 (30)	63 (41)

Data are no. (%), unless otherwise noted. N values <180 are due to missing clinical data. APR, abdominoperineal resection; CAA, coloanal anastomosis; LAR, low anterior resection; NA, not applicable; RT, radiation treatment; SPS, sphincter-sparing surgery; TAE, transanal excision; TEM, transanal endoscopic microsurgery.

a N for anastomosis level are 144 for eligible patients and 126 for SPS patients.

The median time between baseline survey completion and surgery was 0.5 weeks. All patients who were treated with neoadjuvant radiation completed surveys after the completion of radiation. The median duration in the study (from baseline to the last recorded survey) was 111 weeks. This was significantly longer for patients who underwent SPS than for those who did not (median, 70 vs. 49 weeks; p=.001).

### QOL

**Figure 2:**
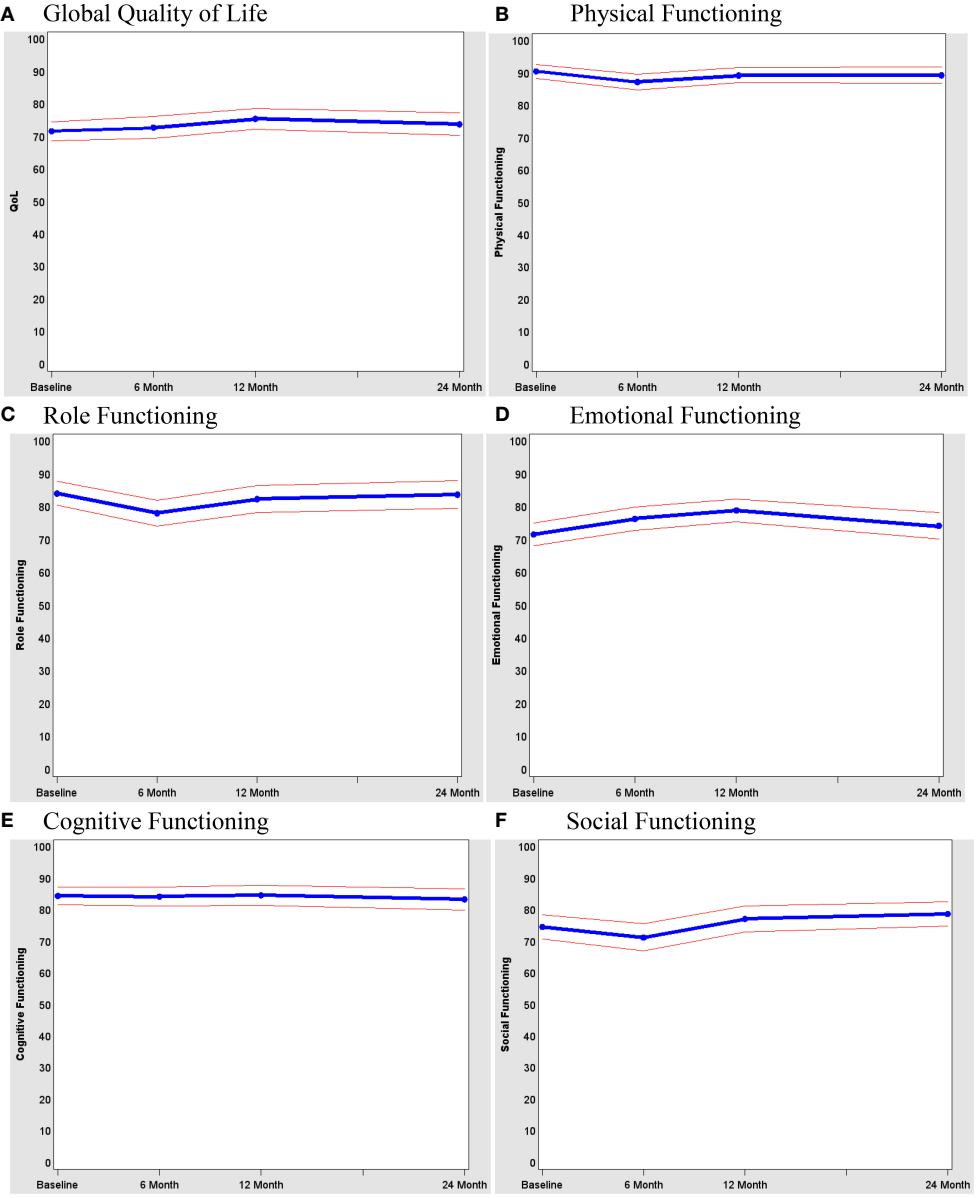
Quality of life of all patients. **(A)** Global Quality of Life. **(B)** Physical Functioning. **(C)** Role Functioning. **(D)** Emotional Functioning. **(E)** Cognitive Functioning. **(F)** Social Functioning.

Baseline and 24-month function and QOL scores are reported for SPS and non-SPS patients in **Table 2**. As shown in **Figure 2**, QOL scores were consistently high; they were lower at 6 and 12 months but, for most patients, returned to baseline levels by 24 months. There were no significant differences in the QOL trend between SPS and non-SPS patients (p=0.29). Among the QOL subscales expected to be most affected by rectal cancer surgery, physical (p=0.03), role (p=0.008), emotional (p=0.002), and social (p=0.003) functions were decreased from baseline.

**Table 2:** Baseline and 24-month function and quality of life scores for the non-SPS and SPS cohorts.

Measure	No SPS				SPS			
	Baseline		24 Months		Baseline		24 Months	
	N	Mean (SD)	N	Mean (SD)	N	Mean (SD)	N	Mean (SD)
Function								
MSK BFI	NA	NA	NA	NA	129	54.6 (7.5)	103	50.8 (9.2)
Frequency	NA	NA	NA	NA	134	22.7 (4.1)	105	22.3 (4.4)
Urgency	NA	NA	NA	NA	134	17.2 (2.8)	105	14.6 (3.7)
Diet	NA	NA	NA	NA	130	14.6 (3.4)	103	13.7 (3.6)
Female Sex Function	3	7.7 (11.2)	1	2.8	44	13.4 (11.3)	32	17.5 (11.1)
Male Sex Function	14	39.4 (28.7)	10	37.1 (23.9)	71	52.8 (21.5)	52	50.2 (22.3)
Micturition	22	24.2 (16.7)	14	23.8 (13.7)	136	18.0 (16.7)	102	15.3 (16.2)
Quality of Life								
Global Health Status	21	65.5 (21.1)	14	72.6 (20.4)	137	73.0 (18.7)	104	74.9 (21.1)
Physical Function	22	83.3 (20.6)	14	84.8 (18.8)	137	91.7 (12.1)	103	91.0 (14.4)
Role Function	22	69.70 (37.0)	14	82.1 (23.9)	136	86.5 (20.3)	104	85.1 (24.0)
Emotional Function	22	65.9 (24.3)	14	78.6 (22.8)	135	72.4 (22.8)	103	73.9 (24.3)
Cognitive Function	22	88.6 (14.9)	14	89.3 (15.5)	136	84.2 (18.3)	104	82.4 (19.4)
Social Function	22	70.5 (30.8)	14	84.5 (22.1)	135	75.1 (23.4)	104	79.0 (24.1)

MSK BFI, Memorial Sloan Kettering Bowel Function Instrument; SD, standard deviation; SPS, sphincter-sparing surgery. NA, Not applicable.

Baseline clinical and demographic variables were examined as predictors of QOL at 24 months (**Table 3**). As expected, low QOL at baseline was strongly related to QOL at 24 months (p<0.001). QOL was negatively affected by the use of radiation (p=0.02) and the type of surgery performed (CAA vs. LAR vs. APR) (p=0.05) and the differences in mean QOL score for each of these variables ranged from 9 to 10 points. On further examination of type of surgery, handsewn anastomoses (vs. stapled) resulted in lower QOL (p=0.04). In a multivariate model that included level of tumor, radiation (yes or no), and type of anastomosis (handsewn vs. stapled), only type of anastomosis was predictive of worse QOL (p=0.03).

**Table 3:** Quality of life univariate and multivariate predictors at 24 months.

Variable	N	Mean (SD)	Median (range)	Univariate p-value	Multivariate p-value
Sex				0.41	
Female	50	75.3 (23.4)	83.3 (0-100)		
Male	67	73.9 (19.5)	75 (16.7-100)		
Age				0.92	
<55	54	73.9 (20.9)	79.2 (16.7-100)		
55-74	52	75.5 (21.0)	83.3 (0-100)		
>75	10	72.5 (24.6)	75.0 (33.3-100)		
Marital Status				0.86	
Single/Widowed	34	75.3 (19.8)	83.3 (16.7-100)		
Partnered	82	74.2 (21.7)	75 (0-100)		
Distance from Anal Verge, cm				0.69	0.71
0-5	20	72.9 (16.4)	75 (41.7-100)		
5-10	70	74.1 (22.9)	83.3 (0-100)		
>10	26	76.6 (19.9)	83.3 (33.3-100)		
Treatment				**0.02**	0.06
Chemo Only	21	82.5 (16.4)	83.3 (41.7-100)		
Chemoradiation	67	70.5 (21.0)	66.7 (16.7-100)		
None	28	78.0 (22.70)	83.3 (0-100)		
SPS- Surgery				0.28	
J-Pouch	32	79.2 (19.6)	83.3 (16.7-100)		
Straight	70	72.7 (21.9)	75.0 (0-100)		
NA	14	72.6 (20.3)	75.0 (33.3-100)		
SPS- Anastomosis				**0.04**	**0.03**
Handsewn	29	65.5 (25.1)	66.7 (0-100)		
Stapled	74	78.4 (18.6)	83.3 (16.7-100)		
NA	14	72.6 (20.3)	75.0 (33.3-100)		
Diversion				0.27	
Ileostomy	77	72.9 (22.2)	75.0 (0-100)		
Colostomy	14	72.6 (20.3)	75.0 (33.3-100)		
None	25	80.3 (17.7)	83.3 (33.3-100)		
Preop Stage				0.37	
I	20	75.8 (21.9)	83.3 (0-100)		
II	19	67.9 (25.8)	66.7 (16.7-100)		
III	67	77.1 (19.0)	83.3 (33.3-100)		
Postop Stage				0.20	
0	37	80.4 (16.8)	83.3 (50-100)		
I	39	70.3 (22.8)	75.0 (0-100)		
II	23	74.6 (23.8)	83.3 (16.7-100)		
III	16	70.8 (21.3)	70.8 (16.7-100)		
IV	1	75	75.0 (75-75)		
Anastomosis Level				**0.01**	
<4 cm	29	67.8 (23.1)	66.7 (0-100)		
≥4 cm	57	79.4 (20.2)	83.3 (16.7-100)		
Surgery				**0.05**	
APR	14	72.6 (20.3)	75.0 (33.3-100)		
LAR	36	81.0 (18.9)	83.3 (33.3-100)		
LAR/CAA	66	71.3 (21.9)	75.0 (0-100)		

APR, abdominoperineal resection; CAA, coloanal anastomosis; LAR, low anterior resection; NA, not applicable; SD, standard deviation. Bold values denote statistical significance at the p < 0.05 level.

### Function

**Figure 3:**
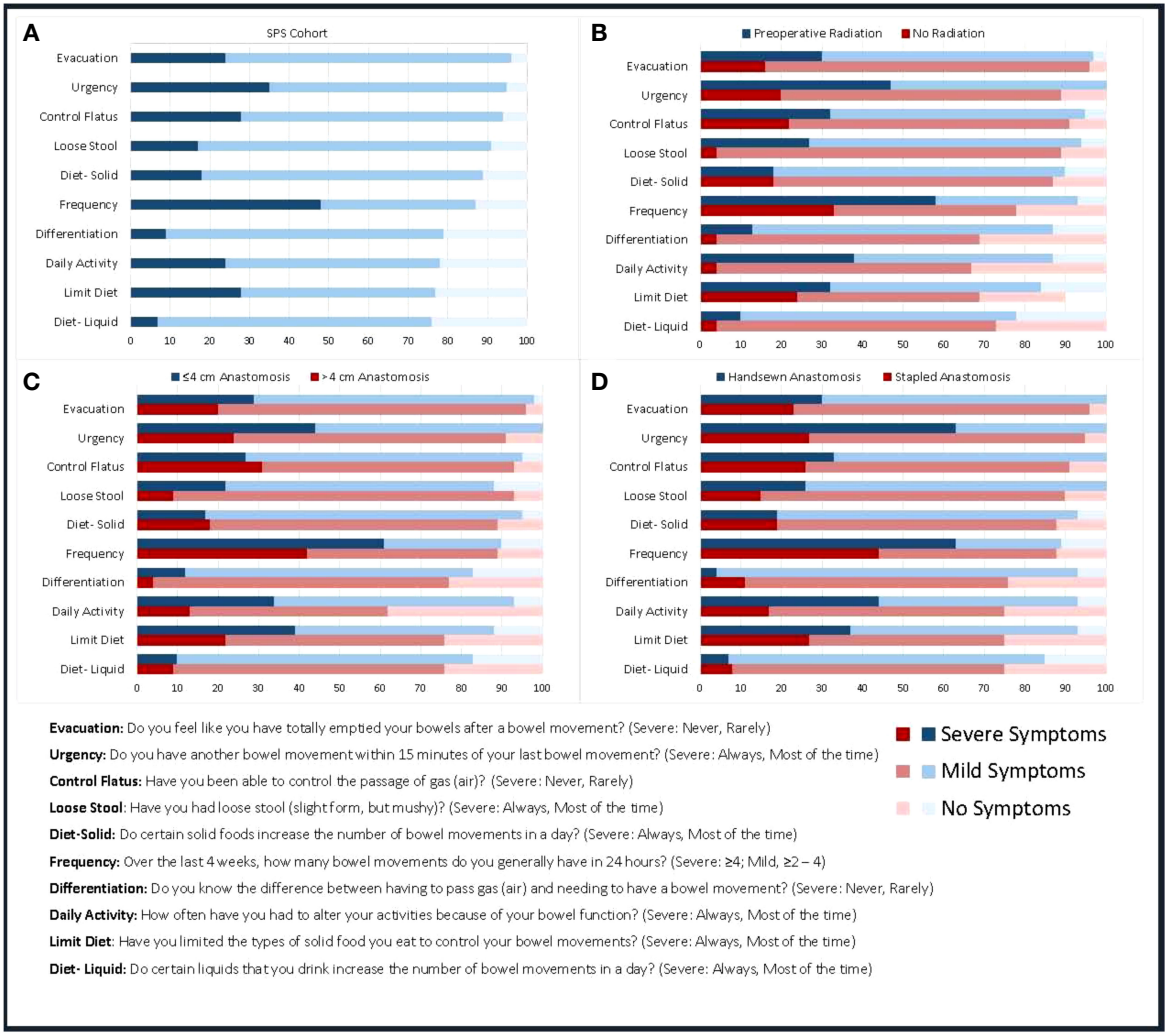
Symptoms reported at 24 months for patients who underwent SPS **(A)**, had anastomoses ≤4 cm and >4 cm **(B)**, had handsewn and stapled anastomoses **(C)**, and underwent preoperative radiation **(D)**.

The scores on the BFI were normally distributed and demonstrated significant differences in demographic and clinically defined groups. As expected, patients with BFI scores in the 10^th^ and 90^th^ percentiles had markedly different symptom profiles, with the most-marked differences in the following measures: not getting to the toilet on time (92% vs. 0%), having soilage during the night (100% vs. 0%), and having to alter daily activities (100% vs. 17%). Bowel function appeared to be significantly impaired. The 10 symptoms most commonly reported on the BFI, and their severity at 24 months—for the whole cohort and for patient subgroups at risk for impaired bowel function on the basis of having low anastomoses (≤4 cm), having handsewn anastomoses, and undergoing preoperative radiation treatment—are shown in **Figure 3**.

**Figure 4:**
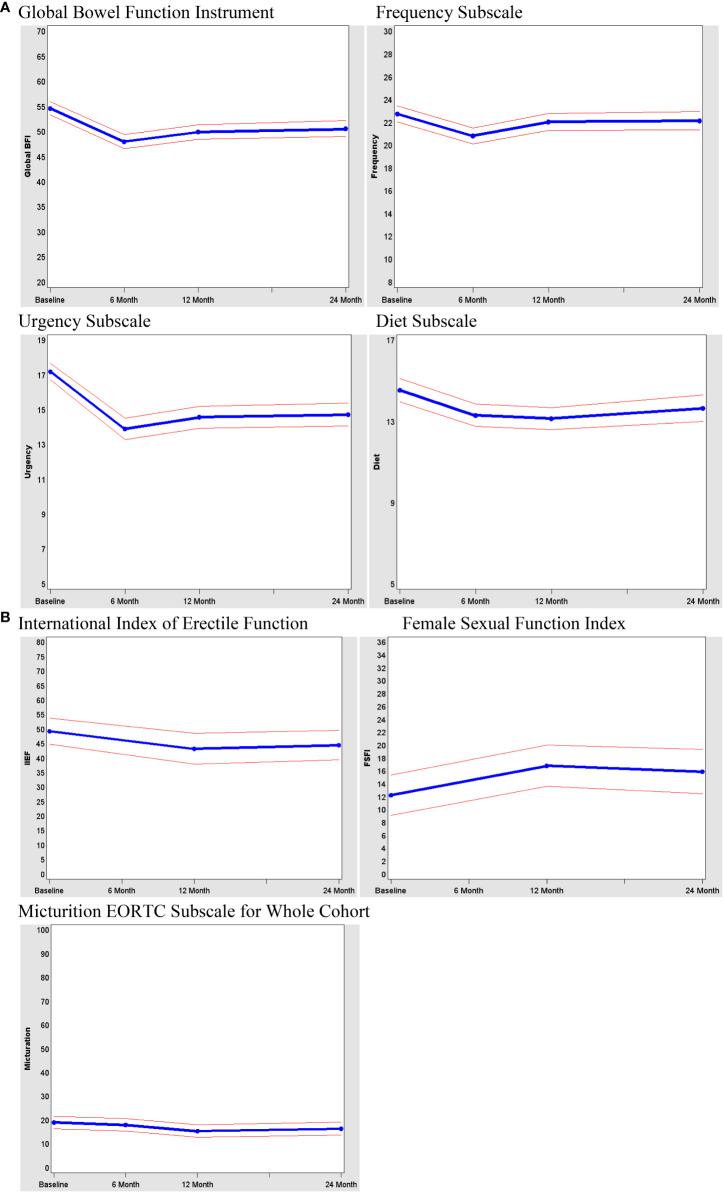
**(A)** Bowel function among patients with sphincter-sparing surgery over time. **(B)** Sexual function and bladder function of all patients.

Longitudinal results among SPS patients show that BFI scores across all subscale domains and total bowel function decreased after treatment for rectal cancer (**Figure 4A**). Bowel function reached a nadir at 6 months and then recovered somewhat by 12 and 24 months, although it never returned to preoperative levels. The domains of frequency and urgency appeared to decline the most at 6 and 24 months.

We evaluated the relationship between bowel function at 24 months and clinical and demographic characteristics at baseline. Radiation had an adverse effect on bowel function (median BFI, 46 vs. 56; p<.001). Distal anastomoses (as measured by LAR vs LAR/CAA, handsewn vs stapled, temporary stoma) also resulted in significantly lower bowel function scores (<5 points) in BFI scores.

Sexual and bladder function were assessed for the entire cohort (**Figure 4B**). In general, sexual function was poor during the study period. From baseline to 24 months, there was minimal improvement in sexual function among women and a decline in sexual function among men. The reason for small improvement in sexual function among women after surgery in this study is not clear and deserves further study, female sexuality is contigent upon complex physical and psychological elements (21). Of importance, the majority of women (77% at baseline vs. 70% at 24 months) and a large proportion of men (27% at baseline vs. 37% at 24 months) met the criteria for sexual dysfunction. Although the numbers were small, there did not appear to be differences in sexual function between patients who underwent SPS and those who did not. Change in bladder function within the cohort was significantly worse (trend p=0.049) from baseline to 24 months, but this change may not be clinically significant. There did appear to be more bladder dysfunction over time in non-SPS patients than in SPS patients (p=0.0085), and this difference appeared to be clinically significant.

### QOL and function

**Figure 5:**
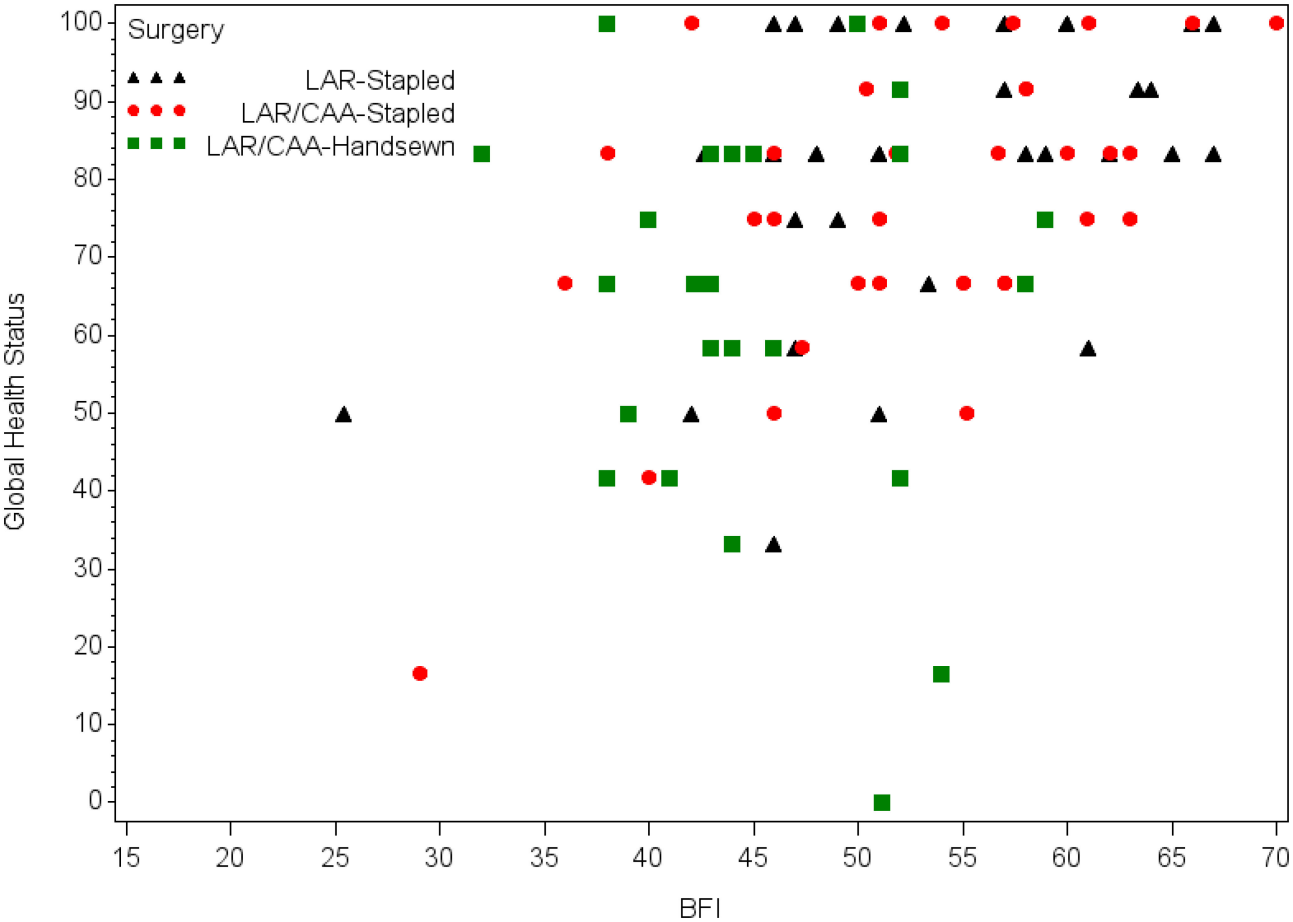
Quality of life (QOL) and bowel function at 24 months (mean QOL for patients with APR = 70). BFI, bowel function instrument; CAA, coloanal anastomosis; LAR, low anterior resection.

To further investigate the relationship between bowel, bladder, and sexual function and QOL, we examined the correlation between these scores (**Figure 5**). Although higher QOL at 24 months was generally associated with higher scores on the BFI at 24 months, there was significant variation. Among SPS patients, bowel function (MSK BFI) at 24 months was correlated with QOL (Pearson correlation, 0.41; p<.0001). In all patients, bladder function at 24 months was correlated with QOL (Pearson correlation, -0.33; p<.0001). Among patients with sexual dysfunction, QOL was significantly lower on the global QOL scale (p=.0015).

## Discussion

In this prospective study, impairment of bowel, bladder, and sexual function was significant and persisted to 24 months, regardless of sphincter preservation. Patients with low tumors were especially at risk for impaired bowel function. In fact, patients with low anastomoses had lower global QOL at 24 months than patients with permanent stomas. For most patients who remained disease free after curative-intent treatment for rectal cancer, QOL returned to baseline levels by 24 months. These findings have important implications for preoperative counseling and surgical decision-making.

Our results corroborate the findings of a similar study performed in Denmark, confirming that low anastomoses are associated with significant fecal urgency and worse QOL than patients with permanent ostomies (22). When the opportunity for sphincter preservation exists, patients may understandably opt to avoid a permanent ostomy and the associated need for lifelong management of an ostomy appliance. This data may help caring physicians to set realistic expectations for these patients by preoperatively outlining the anticipated outcomes up to 2 years, which may empower patients to better cope with the ensuing impairments. Understanding the specific nature of the bowel impairment associated with SPS and its associated loss of QOL can help patients select an operation that is compatible with their lifestyle. Still, ongoing efforts must be made by the clinical team to ensure that quality information is absorbed by patients and that the patient’s needs are considered in the decision-making process.

While our findings can help to improve counseling and decision-making preoperatively, further efforts should be made to improve individual function postoperatively. Evidence concerning the management of patients with poor function after SPS is scarce (23). A recent pilot study in rectal cancer patients after SPS reported that standardized interventions for bowel dysfunction led by trained personnel in specialized clinics may successfully ameliorate symptoms (24). Additionally, transanal irrigation has been receiving increased attention as a treatment option in patients with bowel dysfunction following SPS (25). These efforts may result in improvements in QOL after SPS and are important areas for future research.

Our results reveal considerable heterogeneity among patients. Some patients in our cohort with poor bowel function reported high QOL scores. These “outliers” reflect the clinical impression that some patients continue their activities of daily living without much adjustment, whereas others have to alter their entire lives around their bowel function. In our study QOL improves over time despite persisting dysfunction, likely because of adaptation. Although clinical treatment factors are important predictors of outcomes, the influence of expectations and individual coping style also merit investigation.

An advantage of our study is that it uses a specific bowel function instrument, the MSK BFI, which is anchored to restoration of bowel continuity surgery, rather than from the initial surgery. Additionally, in this study which featured an overall bowel function score, several additional clinical variables—such as level of anastomosis, type of anastomosis, patient sex, and patient age—were also evaluated, making the results especially useful for preoperative discussions about the anticipated outcomes of surgery. Moreover, our response rates were much higher, and spanned a longer period than those previously published, enabling us to better understand the effects of rectal cancer treatment on bowel function (26–28).

Our longitudinal findings clearly demonstrate a persistent deficit in function after treatment. The trajectory of improvement observed in the data suggests that, if after 12 months little improvement is noted, further improvement is unlikely to be seen in the future. Of interest, despite the lack of change in function, QOL improves. This may be because the diagnosis of cancer has a tremendous effect on patients’ QOL and that this effect “dissolves” after treatment. Likely, QOL after rectal cancer therapy is mitigated by several factors. Regardless, there remains a persistent association between QOL and function. While longitudinal cohort studies take a considerable amount of time for data maturation, they are an important part of understanding the long-term effects of function and QOL.

Our study has several limitations. Although the MSK BFI is validated and has domain-specific detail, shorter instruments, such as the Low Anterior Resection Syndrome Score (LARS score) have been published and validated (29). Although the two instruments may measure the same construct, the cohorts used to develop the instruments differed significantly in terms of clinical factors, such as tumor level and surgical techniques. Recent studies have confirmed good correlation and similar discriminant validity of these two questionnaires (30, 31). An additional limitation of our study is that it is a single-center study focused on patients treated by highly subspecialized surgeons who are sought out for their technical skill and their ability to perform sphincter preservation for ultralow tumors, with 26% of SPS anastomoses being handsewn. As such, functional impairment may be overestimated, compared with other studies at centers where sphincter preservation is not performed as often. Additionally, in this study only open surgical technique was used due to oncologic concerns of minimally invasive surgery at the time of study. Whether minimally invasive techniques, such as robotic-assisted surgery offer benefits in functional outcomes and quality of life should be further investigated in future studies The results of this study are still relevant, as adoption rates for minimally invasive rectal cancer surgery remain relatively low compared to other fields of surgery (32). Another limitation of our study includes the use of tumor distance from the anal verge, rather than distance of anastomosis from the anal verge, with the latter described as a more precise predictor of bowel dysfunction (33). This choice was necessary in order to standardize the results, as approximately 18% of patients had missing data regarding exact height of anastomosis performed at time of surgery. Finally, in this patient cohort, a watch-and-wait strategy was not offered as a non-operative alternative to sphincter-preserving surgery. Watch and wait with selective organ preservation for patients with locally advanced rectal cancer has been increasingly adopted, and may lead to better function and QoL when compared with SPS, or permanent stoma (34, 35).

Regardless of its limitations, this prospective study has a long follow-up and high response rates. The exhaustive nature of this clinical information enables its use in the counseling of patients. The careful evaluation of bladder, bowel, and sexual function using validated instruments has further enabled us to understand the interactions between these variables, as well as their relationship to QOL.

In conclusion, impairment of bowel, bladder, and sexual function following rectal cancer treatment is significant and persists regardless of sphincter preservation. Low anastomosis is associated with worse global QOL compared to a permanent stoma. Global QOL returns to baseline levels following successful rectal cancer treatment after 2 years, regardless of sphincter preservation, likely due to adaptation. The findings of this study have the potential to aid physicians when counselling patients with rectal cancer about the long-term effects of function and global QOL associated with surgical treatment.

## Data availability statement

The raw data supporting the conclusions of this article will be made available by the authors, without undue reservation.

## Ethics statement

The studies involving human participants were reviewed and approved by MSKCC IRB. The patients/participants provided their written informed consent to participate in this study.

## Author contributions

All authors: Conception and design, collection and assembly of data, data analysis and interpretation, manuscript writing and approval of final document. All authors contributed to the article and approved the submitted version.

## Funding

This work was supported, in part, by NIH/NCI Cancer Center Support Grant P30 CA008748. Financial support was also provided by a Career Development Award from the American Society of Clinical Oncology.

## Conflict of interest

EP : Travel, Accommodations, Expenses: Intuitive Surgical

SP: Consulting or Advisory Role: ByHeart

LT: https://openpaymentsdata.cms.gov/physician/631475

IW: https://openpaymentsdata.cms.gov/physician/643564

JS: Consulting or Advisory Role: Guardant Health

MWi: Consulting or Advisory Role: Precisca Research Funding: Clinical Genomics Patents, Royalties, Other Intellectual Property: UpToDate Section Editor

GN: Open Payments Link: https://openpaymentsdata.cms.gov/physician/851428 José

GG: Employment: Intuitive Surgical Honoraria: Intuitive Surgical, Myriad Genetics Speakers' Bureau: Intuitive Surgical

MWe: Employment: BridgeBio (I) Stock and Other Ownership Interests: BridgeBio

PP: https://openpaymentsdata.cms.gov/physician/662365

DS: https://openpaymentsdata.cms.gov/physician/389395

JG-A: Stock and Other Ownership Interests: Intuitive Surgical Consulting or Advisory Role: Medtronic, Intuitive Surgical, Johnson & Johnson

The remaining author declares that the research was conducted in the absence of any commercial or financial relationships that could be construed as a potential conflict of interest.

## Publisher’s note

All claims expressed in this article are solely those of the authors and do not necessarily represent those of their affiliated organizations, or those of the publisher, the editors and the reviewers. Any product that may be evaluated in this article, or claim that may be made by its manufacturer, is not guaranteed or endorsed by the publisher.
